# Conduct of vaccination in hard-to-reach areas to address potential polio reservoir areas, 2014–2015

**DOI:** 10.1186/s12889-018-6194-y

**Published:** 2018-12-13

**Authors:** Samuel Bawa, Faisal Shuaib, Mahmoud Saidu, Adamu Ningi, Suleiman Abdullahi, Bashir Abba, Audu Idowu, Jibrin Alkasim, Kulchumi Hammanyero, Charity Warigon, Sisay G. Tegegne, Richard Banda, Charles Korir, Yared G. Yehualashet, Tesfaye Bedada, Chukwuji Martin, Peter Nsubuga, Usman S. Adamu, Bassey Okposen, Fiona Braka, Alemu Wondimagegnehu, Rui G. Vaz

**Affiliations:** 1 World Health Organization, Country Representative Office, Abuja, Nigeria; 2 National Primary Health Care Development Agency, Abuja, Nigeria; 3 Global Public Health Solutions, Atlanta, GA USA; 4 National Polio Emergency Operation Center, National Primary Health Care Development Agency, Abuja, Nigeria

**Keywords:** Vaccination, Hard-to-reach, Low population immunity for polio

## Abstract

**Background:**

The Global Vaccine Action Plan (GVAP) seeks to achieve the total realization of its vision through equitable access to immunization as well as utilizing the immunization systems for delivery of other primary healthcare programs. The inequities in accessing hard-to-reach areas have very serious implications for the prevention and control of vaccine-preventable diseases, especially the polio eradication initiative.

The Government of Nigeria implemented vaccination in hard-to-reach communities with support from the World Health Organization (WHO) to address the issues of health inequities in the hard-to-reach communities. This paper documents the process of conducting integrated mobile vaccination in these hard-to-reach areas and the impact on immunization outcomes.

**Methods:**

We conducted vaccination using mobile health teams in 2311 hard-to-reach settlements in four states at risk of sustaining polio transmission in Nigeria from July 2014 to September 2015.

**Results:**

The oral polio vaccine (OPV)3 coverage among children under 1 year of age improved from 23% at baseline to 61% and OPV coverage among children aged 1–5 years increased from 60 to 90%, while pentavalent vaccine (penta3) coverage increased from 22 to 55%. Vitamin A was administered to 78% of the target population and 9% of children that attended the session were provided with treatment for malaria.

**Conclusions:**

The hard-to-reach project has improved population immunity against polio, as well as other routine vaccinations and delivery of child health survival interventions in the hard-to-reach and underserved communities.

## Background

The Global Vaccine Action Plan (GVAP) seeks to achieve the total realization of its vision, in which all individuals and communities enjoy the full benefit of immunization, through equitable access to immunization as well as utilizing the immunization systems for delivery of other primary healthcare programs [1]. The inequities in accessing hard-to-reach areas have very serious implications for the control and prevention of vaccine-preventable diseases, especially the polio eradication initiative. Hard-to reach is a term used to describe those subgroups of the population that may be difficult to reach or involve in public health programs, especially with regards to health and social inequalities [2–4]. In this context, the project operationally defined hard-to-reach areas as geographically difficult terrain, with any of the following criteria: having inter-ward, inter-Local Government Area (LGA), or interstate borders, scattered households, a nomadic population, or a waterlogged/riverine area, with no easy access to healthcare facilities and insecurity.

Hard-to-reach areas serve as a niche for sustaining the transmission of diseases. Children in remote areas mostly have low immunization coverage, and this gap impacts child health [5]. The National Polio Eradication Emergency Plan (NPEEP) 2014 reported that, in Nigeria, over a third of cases of polio in 2013 were under-immunized and from the hard-to-reach settlements in the four polio-endemic states of Bauchi, Borno, Kano, and Yobe. The plan recommended targeting the hard-to-reach to improve population immunity and interrupt polio transmission as one of its strategic priorities for 2014 [6].

Reaching the hard-to-reach takes time due to poor or nonexistent roads, difficult terrain, and long distances to travel. Given the need to reach these communities with the polio vaccine, and the time and resources required and the additional health needs in these areas of Nigeria, the mobile outreach of the reaching every ward/district (REW/RED) was adopted. The REW strategy provides evidence of an improvement in the delivery of routine immunization services as well as strengthening the immunization delivery system to meet the need for integrated delivery of other child and maternal survival interventions [7]. Furthermore, the traditional immunization service delivery system is not sufficient for rural, hard-to-reach areas [8]. Vaccination in combination with other interventions has been shown to improve child survival outcomes [9, 10]. The Government of Nigeria implemented vaccination in hard-to-reach communities with support from the World Health Organization (WHO) to address the issues of health inequities in the underserved communities where, often, the population immunity for polio is inadequate. This paper documents the process of conducting integrated mobile vaccination in hard-to-reach areas and its impact on immunization outcomes.

## Methods

**Fig. 1:**
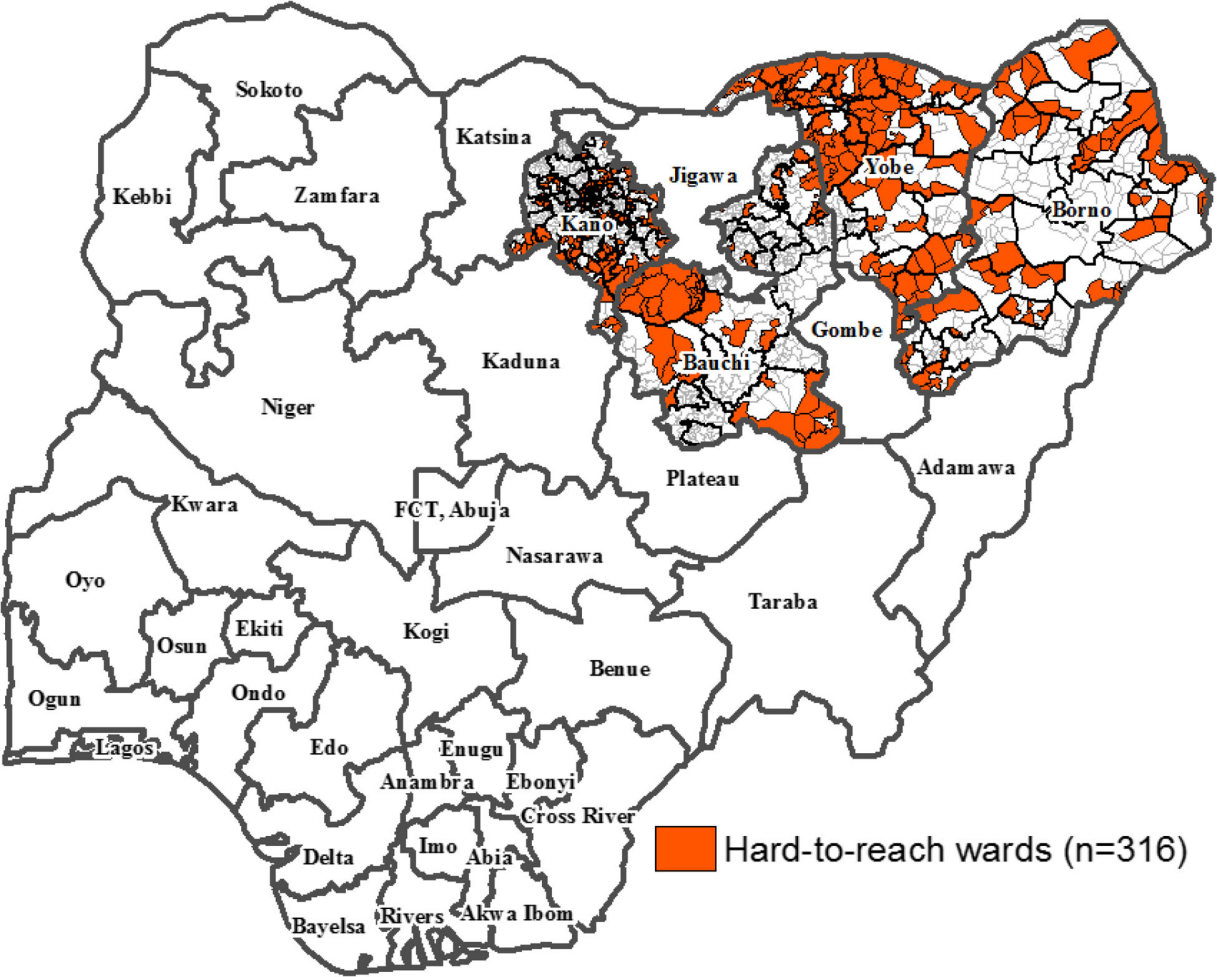
Map of Nigeria showing the hard-to-reach project states and wards, 2014–2015

We conducted vaccination in hard-to-reach populations in 2014–2015. Using epidemiologic and program criteria (i.e., analysis of the supplemental immunization activities (SIAs) micro-plan settlement profile data), the polio program selected 2311 hard-to-reach settlements in the four states which were at risk of sustaining wild poliovirus (WPV) transmission in 2014. Of these, 763 were in Bauchi, 620 in Borno, 406 in Kano, and 522 in Yobe states (Fig. 1).

### Planning and development of mobile session plans

In the initial planning stages, we paid advocacy visits and convened planning meetings with host government policymakers to embrace and support the hard-to-reach project. We conducted micro-planning and micro-census of the targeted population in the selected hard-to-reach settlements with validation, dropping those settlements that did not fulfill the inclusion criteria of being hard-to-reach. Also, the number of mobile teams was determined based on the target population of mothers and children under 5 years of age in the selected hard-to-reach settlements covered in the state and LGA (a mobile team workload was 100 clients per session per day) and catchment health facilities of the selected hard-to-reach settlements. Furthermore, session plans and days of activity were defined based on distance from the take-off points to each settlement and the most appropriate transport required, with input from the community leaders.

### Community linkage and engagement of community structure

The community was engaged from the micro-planning process stage to the identification of community members who served as mobilizers and town announcers in the settlements. The mobilizers were sensitized on their roles in informing and mobilizing the clients on the mobile session days, crowd control during sessions, and immunization defaulter tracking.

### Determination and procurement of commodities for intervention

Essential drugs and commodities were procured and distributed based on estimates of the actual needs of the target population. The supply was procured centrally and distributed to the LGAs on a monthly basis, and then on to the mobile health teams on a weekly basis. A logistic management system was established that ensured adequate supply was maintained at all levels. The logistic management system was also reviewed according to the morbidity or consumption pattern of commodities and supplies at the mobile team level.

### Human resources requirement

A social mobilization and communication specialist and national logistician were engaged at the national level and, at the state level, four cluster consultant logisticians, four state data assistants, four store assistants, and 55 LGA facilitators (LGAFs) were recruited. Sixty-five mobile outreach teams were mobilized, with each team made up of four health personnel (nurse-midwife, senior community health extension worker, community health extension worker and health records assistant). The LGA hard-to-reach focal persons, routine immunization focal persons, community mobilizers, and town announcers were also involved in supporting these teams.

### Development of guidelines, training manual, and information education and communication (IEC) materials and devices

We developed a hard-to-reach project training manual and used it for the training of teams, and a standard operating procedure was also developed to guide the implementation process of vaccination in the hard-to-reach areas. A new training package was also developed for volunteer community mobilizers (VCMs) with IEC materials adapted to enable them to mobilize the communities.

### Baseline assessment of the hard-to-reach settlements

We conducted an assessment of the baseline situation of routine immunization coverage, access, and coverage of child and maternal health services, health personnel, health knowledge, attitudes, and health-seeking behaviors in the LGAs. The availability of a cold chain, data management, and IEC materials were also appraised. The analysis of the assessment provided evidence-based and context-specific programmatic and communication information that helped to prioritize interventions to reach children and women in hard-to-reach settlements.

### Data management

We adapted the data tools for routine immunization and other vaccination activities to capture information and harmonized with the National Health Management Information System (NHMIS) [11]. The hard-to-reach mobile team documented all services provided to clients, including oral polio vaccine (OPV) administered to children aged 12–59 months during the session. The children administered OPV were thumb marked with indelible pen marker.

All information collected was summarized after each session, and the mobile data captured were transmitted immediately before leaving the last settlement visited. The catchment area routine immunization focal person in attendance at the session also transferred the same information into the routine immunization (RI) data management tools of the respective health facility. The teams submitted reports on a daily and weekly basis to the LGA through the LGAF, who compiled and submitted results for the entire week. Every child immunized was issued a child health card and all pregnant women who attended to received antenatal clinic cards. All commodities and treatment provided to clients were based on the minimum package for primary healthcare (PHC) and referral system to higher levels of care in Nigeria [12].

### Monitoring, supportive supervision, and geographic information system (GIS) tracking of teams

We engaged hard-to-reach LGAFs to monitor the activities of the hard-to-reach teams in the LGAs. They were also supported by the WHO polio eradication initiative surge capacity personnel, alongside local government officials. Theses provided weekly supervisory support and monitored visits and documented the activities using a standard checklist. The checklists were uploaded on to an open data kit (ODK) using a mobile data device (android phone) and used to generate reports [13], tracked through real-time transmission, with support from e-Health Nigeria who regularly provided feedback information to WHO.

The enhanced mobile outreach strategy was used to provide routine immunization with a basic integrated package of primary healthcare interventions focused on maternal, newborn, and child health. These interventions addressed the main causes of child mortality (i.e., pneumonia, diarrhea, malaria, and vaccine-preventable disease) and provided preventive services such as iron folate and malaria prevention for pregnant women. The project’s intent was not research and, as such, it did not go through an ethical clearance process with the Ministry of Health. However, Government permission and buy-in was granted.

## Results

The hard-to-reach intervention was implemented in 2311 preselected, nonurban, hard-to-reach and underserved communities in 316 wards in 67 very high-risk LGAs in Bauchi, Borno, Kano and Yobe states (Table 1). The mobile teams submitted reports of the sessions conducted on a weekly basis and also transmitted real-time information capturing the time that sessions were started and completed, and the geographic location of the settlement where sessions were conducted.

**Table 1:** Distribution of hard-to-reach settlements and target populations, 2014–2015

				Children				
State	LGAs	Wards	Settlements	0–11 months	12–59 months	0–59 months	Pregnant Women	Women of childbearing age (15–49 years)
Bauchi	9	49	763	10,502	48,232	70,069	7931	52,409
Borno	17	73	620	12,019	44,320	67,572	11,504	54,058
Kano	27	109	406	5154	35,662	40,525	3652	32,420
Yobe	14	85	522	8562	34,925	44,713	4516	35,682
Total	67	316	2311	36,237	163,137	222,879	27,603	174,569

The OPV3 coverage among children under 1 year old showed an upward trend within one quarter in all the states, with Borno having the highest coverage of 80% in the first quarter (Table 2). The overall cumulative coverage for all the states was 38% for the first quarter of implementation, July to September 2014. By the second quarter of implementation (October to December 2014), all the states showed improvement in OPV3 coverage to 48, 44, and 47% for Bauchi, Borno, and Yobe states, respectively, except for Kano which reported only 18%. The OPV3 coverage by the July to September 2015 quarter was 65% for Bauchi, 61% for Borno, 73% for Kano, and 44% for Yobe (Table 2).

**Table 2:** Trend in percentage coverage of oral polio vaccine (OPV)3, the third pentavalent vaccine (penta3), and measles in children under 1 year of age in the hard-to-reach settlements, 2014–2015

	Baseline	July–Sept 2014			Oct–Dec 2014			Jan–Mar 2015			Apr–Jun 2015			Jul–Sept 2015		
State	OPV3	OPV3	Penta3	Measles	OPV3	Penta3	Measles	OPV3	Penta3	Measles	OPV3	Penta3	Measles	OPV3	Penta3	Measles
Bauchi	21	36	28	116	48	41	123	74	55	111	77	54	112	65	47	92
Borno	27	80	36	95	44	31	57	46	25	66	61	47	88	61	55	72
Kano	15	17	9	84	18	16	31	39	39	23	167	166	68	73	73	22
Yobe	27	18	6	85	47	42	48	51	46	51	40	31	61	44	41	45
Total	23	38	20	95	39	33	65	53	41	63	86	75	82	61	54	58

**Fig. 2:**
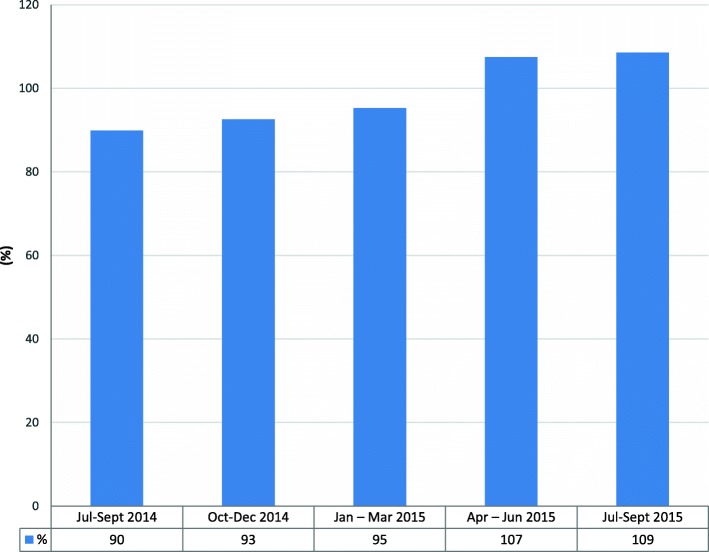
Trend of percentage OPV coverage in children under 5 years of age in hard-to-reach settlements, 2014–2015

Targeting children aged 1–5 years with OPV showed an increasing trend (Fig. 2); it had consistently gone beyond 90% for the period of implementation, with coverage above 100% in April to June and July to September 2015, due to influx of more clients from outside the planned catchment areas to access treatment, which was provided free of charge.

The coverage of the third dose of the pentavalent vaccine (penta3) was very low in all the states during the first quarter of implementation (6% in Yobe and 9% in Kano states; Table 2), but increased to 41 and 73%, respectively, by September 2015. Overall, the penta3 coverage increased from 20% at baseline to 54% by September 2015 across all the implementing states.

**Fig. 3:**
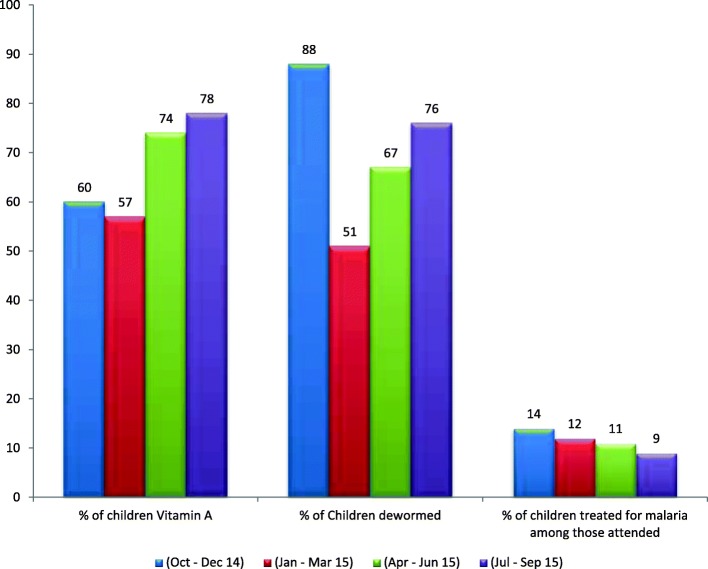
Trend of vitamin A, deworming, and those treated for malaria among children under 5 years of age in hard-to-reach settlements, 2014–2015

Vitamin A, albendazole (an antihelminthic agent), and malaria treatment were administered to children under 5 years old (Fig. 3). Of the targeted children, 78% were provided with vitamin A in July to September 2015, an increase compared with the earlier quarters. The proportion of children dewormed, however, fluctuated between the different quarters of implementation with the highest recorded in October to December 2014. There was a decreasing trend of children provided with treatment for malaria in the period under review. This ranged from 14 to 9% from October–December 2014 to July–September 2015. A rapid diagnostic test (RDT) for malaria was introduced as part of the roll-back-malaria treatment policy of prescription of antimalarials to only those who tested positive for the malaria parasite.

A total of 24 cases of acute flaccid paralysis (AFP) were reported from the hard-to-reach settlements over the period of implementation (Table 3). Investigations were conducted, and stool specimens were collected and sent to the national polio laboratory, but all the result were confirmed to be nonpolio AFP.

**Table 3:** Distribution of acute flaccid paralysis (AFP) cases reported from hard-to-reach settlements, 2014–2015

State	2014			2015			Total AFP cases
	AFP cases	WPV	cVDPV	AFP cases	WPV	cVDPV	
Bauchi	0	0	0	5	0	0	5
Borno	2	0	0	4	0	0	6
Kano	5	0	0	6	0	0	11
Yobe	2	0	0	0	0	0	2
Total	9	0	0	15	0	0	24

*cVDPV* circulating vaccine-derived poliovirus, *WPV* wild poliovirus

## Discussion

We found a steady increase in OPV3 and penta3 vaccine coverage in the hard-to-reach areas during the period of implementation of mobile health sessions. The coverage of supplemental doses of OPV to children up to 5 years of age also improved. This finding agrees with those from Bangladesh and India where combined interventions improved the child immunization coverage in rural hard-to-reach areas [5, 14]. The package of intervention provided included services and commodities that addressed the unmet health needs of the communities, which created demand and led to overwhelming acceptance and support for the mobile health teams during the session, despite being previously noncompliant to polio vaccination teams [15].

An increasing number of children received vitamin A and antihelminths, while pregnant women were provided with intermittent preventive therapy (IPT) with an antimalarial. Similarly, the finding of the strategies to reproductive, maternal and child health in difficult to access mountainous locations show that there is benefit for comprehensive, regular outreach teams for service delivery and coverage. Integrated care, community health workers and regular supply of commodities immensely reduce morbidity and mortality of children and women.

We also found that the mobile teams conducted active searches and reported AFP cases from the hard-to-reach settlements. All the cases revealed nonpolio AFPs, in contrast to the NPEEP report where over a third of the polio cases in 2013 were from hard-to-reach settlements [6].

Although the interruption or absence of WPV in the hard-to-reach might not be attributed to the hard-to-reach interventions alone, it is worthy of note that these interventions have also contributed towards improved maternal and child health indices. As in other studies documenting an improvement in routine immunization programs, this study was not conducted using standard research protocols since research was not the primary purpose of the work. The reaching the hard-to-reach project was an intervention to improve population immunity that integrated immunization and other maternal and child health services [16].

Real-time reports from the teams improved accountability and ensured that they conducted the sessions according to plan. Although it may not translate to improved coverage and population immunity, this served as a monitoring and accountability tool which ensured that the sessions were held as planned [17, 18].

Although we did not determine the cost per session for the hard-to-reach in this study, the intervention had the benefit of delivering vaccines and other interventions to areas that would have continued to be missed, with a possible consequence of polio outbreaks. The outreach also provided an opportunity for clients from neighboring settlements not included in the initial plan to benefit from these services.

Although the hard-to-reach activities improved OPV and other child survival intervention coverage, a cost-benefit analysis of the intervention should be conducted. The outcome, regarding the benefit and lessons learnt, could be used as an advocacy tool to encourage the government to buy-in to integrated mobile outreach and strengthen primary health care in the states.

Hard-to-reach areas will continue to exist, and their underserved populations are vulnerable to diseases. Mobile outreach to these communities should be established and integrated into the routine immunization outreach service delivery plans, or it could be expanded into a more integrated and well-financed mobile (motorized) outreach program, where the minimum health package could be provided. The hard-to-reach strategy should also be aligned with the one primary healthcare center (PHC) per ward approach in the medium- to long-term planning. Furthermore, government at respective levels should be involved in the planning phase to ensure buy-in and sustainability of the outreaches to the hard-to-reach areas.

The hard-to-reach project has improved population immunity against polio in the hard-to-reach and underserved communities and also improved the delivery of other child and maternal health survival interventions. Ultimately, there should be improved and sustained coverage of the target population with the specified interventions. Furthermore, it has improved reach, addressed inequity to healthcare access to underserved communities, and contributed in interrupting transmission in the states at high risk for polio in Nigeria.

## Conclusions

The hard-to-reach project was implemented to address gaps in population immunity among the inaccessible communities in communities at high risk for polio. Using this integrated approach, routine vaccines and other child health survival interventions were provided to the hard-to-reach and underserved communities. This contributes to polio eradication, health indices, and universal health coverage.

## Abbreviation

AFP: Acute flaccid paralysis
GIS: Geographic information system
GVAP: Gobal Vaccine Action Plan
IEC: Information education and communication
LGA: Local Government Area
LGAF: Local Government Area facilitator
NHMIS: National Health Management Information system
NPEEP: Nigeria Polio Eradication Emergency Plan
ODK: Open data kit
OPV: Oral polio vaccine
Penta: Pentavalent
PHC: Primary healthcare
RED: Reaching every district
REW: Reaching every ward
SIA: Supplemental immunization activity
VCM: Volunteer community mobilizer
WHO: World Health Organization
WPV: Wild poliovirus
